# The Impact of the COVID-19 Pandemic on Romanian Postgraduate Periodontal Residency Teaching: Past Experience, Present Imperatives and Future Considerations in a Multicentric Evaluation

**DOI:** 10.3390/ijerph19084488

**Published:** 2022-04-08

**Authors:** Cristina Andrada Costea, Dora Maria Popescu, Alexandra Roman, Ștefan-Ioan Stratul, Petra Șurlin, Marius Negucioiu, Iulia Cristina Micu, Andreea Ciurea, Patricia Ondine Lucaciu, Luminița Lazăr, Doina Elena Mircioagă, Andrada Soancă

**Affiliations:** 1 Department of Periodontology, Faculty of Dental Medicine, Iuliu Haţieganu University of Medicine and Pharmacy Cluj-Napoca, Victor Babeş St., No. 15, 400012 Cluj-Napoca, Romania; costea.cristinaandrada@yahoo.com (C.A.C.); veve_alexandra@yahoo.com (A.R.); i.cristina.micu@gmail.com (I.C.M.); andreea_candea@yahoo.com (A.C.); andrapopovici@gmail.com (A.S.); 2 Department of Periodontology, Faculty of Dental Medicine, University of Medicine and Pharmacy from Craiova, 2 Petru Rares St., 200349 Craiova, Romania; dora.popescu@umfcv.ro (D.M.P.); surlinpetra@gmail.com (P.Ș.); 3 Department of Periodontology, Faculty of Dental Medicine, Anton Sculean Research Center for Periodontal and Peri-Implant Diseases, Victor Babeș University of Medicine and Pharmacy Timișoara, Bulevardul Revoluției din 1989, No. 9, 300230 Timișoara, Romania; sbs@online.ro; 4 Department of Prosthodontics, Faculty of Dental Medicine, Iuliu Haţieganu University of Medicine and Pharmacy Cluj-Napoca, Clinicilor St., No. 32, 400006 Cluj-Napoca, Romania; sica5319@yahoo.de; 5 Department of Oral Health, Faculty of Dental Medicine, Iuliu Haţieganu University of Medicine and Pharmacy Cluj-Napoca, Victor Babeş St., No. 15, 400012 Cluj-Napoca, Romania; 6 Department of Periodontology, Faculty of Dental Medicine, George Emil Palade University of Medicine, Pharmacy, Science, and Technology of Târgu Mureș, Str. Gheorghe Marinescu Nr. 38, 540139 Târgu Mureș, Romania; 7 Department of Physical Education, University Sport Research Center for Evaluation of Fitness Level–CUSENF, Victor Babeș University of Medicine and Pharmacy Timișoara, Eftimie Murgu Square, No. 2, 300041 Timișoara, Romania; doina.mircioaga@umft.ro

**Keywords:** COVID-19, clinical teaching, e-learning, simulation, periodontology

## Abstract

The aims of this study were to identify the challenges in periodontology postgraduate residency programs during the COVID-19 pandemic by identifying the modifications of educational instruments, to evaluate the impact of hybrid education on periodontology postgraduate programs in terms of resident-centred outcomes, and to evaluate the education efficiency of an innovative teaching approach. Resident doctors from three Romanian dental faculties were included in study groups based on the intensity of clinical training. A web-based questionnaire was used to collect information on residents’ perception about teaching activity. Important educational changes were identified. Moreover, residents learned a periodontal procedure through online training and then performed it on preclinical models three times. The working times were recorded. Statistical analysis was performed. Resident doctors were unsatisfied with clinical practice during the pandemic year, but they positively valued the development of online courses. Learning efficiency improved by repeating the same procedure on preclinical models, as proved by the significant decrease of the working times. E-learning was appreciated as an important component of the new hybrid teaching approach. Reorganization and further emphasis on both preclinical and medical practice, targeted to aid residents perform more accurate and efficient procedures, are recommended.

## 1. Introduction

Due to the severe impact of the COVID-19 pandemic on higher education institutions around the world, in March 2020 medical universities were also forced to interrupt their traditional onsite teaching and practical activities and, in response, transfer them almost entirely to “virtual platforms” of academic study and e-learning [1,2]. Among the dental schools included in an early survey (March–April 2020) of the Association of Dental Education in Europe (ADEE), 90% reported employing online educational software tools, with 72% using live or streamed videos, resorting to links to various online materials (48%) or participating in virtual meetings (65%) and working groups [3]. However, the United Nations Educational, Scientific and Cultural Organization (UNESCO) and a large part of the media expressed high levels of concern regarding the impact of this new, virtual model of instruction on student education [4]. For dental schools in particular, the clinical component posed a major and urgent challenge throughout their adaptation to remote learning strategies. The cessation of clinical dental work during lockdown was imposed as dental professionals were placed at especially high risk of contracting COVID-19 given the unique nature of dentistry, which exposes practitioners to potentially hazardous COVID-19-containing aerosols generated through current procedures [5]. To et al. recognized saliva as a reservoir of SARS-CoV-2 in infected individuals [6]. Fundamental dentistry devices associated with routine care delivery, such as the air-driven high-speed handpiece, the air polisher, the air-water syringe and prophylaxis angles [7,8] were shown to be important aerosol-contaminant drivers. Also, periodontal procedures are always performed in the presence of blood and saliva and rely mostly on the use of ultrasonic scalers, which create the greatest amount of aerosol contamination [9], increasing the overall risk of exposure.

In addition, dental schools have had to manage several activity areas impacted by the prolonged COVID-19 crisis: online teaching, new teaching content using digital resources, implementing online assessment methods, personal development plans for staff (virtual skills, medical and research activities), medical activity management, resident support and self-care.

Although online lectures and e-learning tools have been part of a general common strategy adopted by dental schools, the early management of COVID-19 effects on dental residency programs has been approached in different ways across different universities and specialties. In dental hospitals, clinical work was mainly performed by the senior staff (96%), with some participation of postgraduate students (30%) and a small-scale implication (11%) of undergraduate students in non-clinical activities [3].

As in other EU countries promoting periodontology as a speciality, the Romanian training curriculum for periodontology residency programs consists of 3-year full-time study (6 semesters), corresponding to 180 European Credits (ECTS) broken down into 120 ECTS for academic content and 60 ECTS for clinical learning. Postgraduate residency programs in periodontology develop a curriculum provided by the Romanian Ministry of Health and at the same time follow the standard quality criteria of national (Romanian Agency for Quality Assurance in Higher Education, ARACIS) and EU authorities (European Federation of Periodontology, EFP). Faced with unprecedented challenges during the pandemic, periodontology departments from the three major Romanian medical universities coordinating more than 100 resident doctors had to respond in active and innovative ways to continue the periodontology postgraduate education of the future specialists in periodontology. Residency program coordinators agreed to define the new “good” of the teaching process for postgraduate residency education in periodontology—an eminently practical specialty- and technology-enhanced approach to teaching, all within a week, especially during the lockdown period. A “new” normal had to be adopted at all levels through innovative pedagogy responsive to the standards of the educational process in periodontology. Initially, after the transition to emergency remote teaching, the ultimate goal was to save the academic year. Gradually, all stakeholders involved in the educational process acknowledged the need to target the theoretical and practical skills provided in the curriculum independently of the stay-at-home period with minimal damage to achievement standards.

After the lockdown, a “new” hybrid learning approach based on a combination of online, preclinical, as well as clinical teaching was implemented as a temporary measure that, against all expectations, was subsequently extended due to the threatening infectious transmission. Therefore, more than a year since the onset of the pandemic, it becomes important to reconsider the complex and innovative computer-based teaching approaches applied in periodontology residency programs in terms of learning efficiency and education advancement, in order to validate a spectrum of techniques which can still be provided virtually. The shift to e-learning required an enormous mobilization of human and technical resources and it is considered to have induced several significant benefits to the educational process. Thus, analysing the effects of this transition to e-learning from the resident perspective in order to identify its strengths and limitations becomes necessary. In this respect, we believe that self-reports could be employed as an efficient and attainable means of assessing learning efficiency, teaching performance or the impact of technology use on instruction [10].

Previous literature providing information on the impact of the COVID-19 pandemic on dental medicine focused primarily on medical activity in terms of improving protocols of risk management, evidence-based treatments, or the efficiency of workflows through data sharing, machine learning, artificial intelligence, and blockchain technology [11,12,13]. Moreover, some data on undergraduate dental education have been available [3,14,15,16]. To our knowledge, research results specifically addressing the issue of dental education for residency postgraduate programs have not been reported yet [14].

The first aim of this study was to identify the various challenges of periodontology postgraduate residency programs occurring during the one-year COVID-19 crisis by identifying the modifications of educational and technical instruments. Another aim was to evaluate the impact of periodontology postgraduate hybrid education represented by the e-learning process addressing theoretical and practical subjects, preclinical training and medical activities on resident-centered outcomes, measured through a self-reported questionnaire. Additionally, our study aimed to evaluate the educational efficiency of an innovative teaching approach targeting the reduction of the working time of a periodontal manoeuvre. Depending on the year of study, residents were included in one of the two educational variants: intensive on-line learning and practical training based mostly on pre-clinical approaches, or a less intense on-line program associated with mostly onsite practical medical education. For the purpose of this study, the following null hypothesis was tested: the perceptions of the two groups of resident doctors on the postgraduate educational program did not differ.

## 2. Materials and Methods

### 2.1. Study Design and Sample

A cross-sectional study was conducted using a multicentric approach that included resident doctors enrolled in a periodontology program at the dental faculties of Cluj-Napoca, Timișoara, and Craiova. The three Romanian dental faculties involved in this study were selected based on similarities in curricular content and teaching approaches. The study was carried out between July 2021 and August 2021, and it received ethical approval from an accredited committee of the three universities (203/03.06.2021; 116/19.07.2021; 32/30.06.2021). A firm action plan was perfected by the three periodontology departments to coordinate the complex activities of their residency programs involving online teaching, preclinical training/clinical activity, and administrative actions by transforming former learning activities and implementing new teaching strategies and approaches adapted to respond to the pandemic conditions. The study identified important educational changes developed over a period of 15 months.

Moreover, resident doctors were invited to complete a web-based survey designed to determine the resident-centred impact of the modified format of the teaching schedule during the COVID-19 pandemic through an adapted self-report questionnaire previously validated by our team [17]. The invitation to complete the survey and a questionnaire link were distributed to all residents via e-mail and/or resident online discussion rooms. All residents were voluntarily involved in this study and provided informed consent through the online application in accordance with the EU General Data Protection Regulations (UE) N° 2016/679 prior to survey completion. All participants were informed that no personal data was collected and agreed to complete the survey anonymously.

The resident doctors from the three universities participating in the study were divided into two major groups. The two groups had the same online course activity, but involvement in clinical practice varied. The PARO 1 group included first year residents focused mostly on intensive systematic online teaching of the practical manoeuvres, followed by preclinical practical training on phantoms. The PARO 2 group included senior residents mostly involved in clinical activity and, occasionally, in preclinical training.

The present study also considered the impact of an innovative teaching approach implemented to prepare residents for shorter contact with patients in clinics by increasing learning efficiency in preclinical settings in terms of time saving: a periodontal manoeuvre—a full-mouth periodontal examination—was taught to residents during an online preclinical training and afterwards it was performed three times on different days in order to observe the evolution of the procedure duration. The working time was registered, and the results were assessed comparatively.

### 2.2. Questionnaire on Teaching Activity

A 32-item questionnaire was used to collect data on the perception of periodontology residents towards teaching activity during the COVID-19 crisis. The questionnaire was adapted by four senior periodontologists using the template of a previously validated questionnaire administered to determine student perception and developed in Romanian by a member of our team [17]. Five questions were replaced from the original document in order to more specifically target resident activity and, thus, the Romanian first version of the questionnaire for residents was created.

The overall comprehension of the questionnaire, the graphic presentation, and time to completion were firstly assessed onsite by 20 residents not included in the study. Collected feedback data were used to revise the questionnaire and develop the Romanian second (final) version of the residents’ questionnaire (Appendix A).

The Romanian final version of the questionnaire was compiled by team members and set up using Google Forms.

The first part of the questionnaire collected demographic information (gender, age, year of study) used to describe the study population. Age groups covered the intervals 27–30 years, 31–40 years, and 40 years and over. For the first interval, age was recorded annually.

The questions (Q) evaluating the resident-centred impact of teaching activity during the COVID-19 pandemic were divided into 7 parts, evaluating: (1) technical support (Q1 to Q3), (2) the impact of traffic restrictions and of self-isolation (Q4 to Q7), (3) the impact of online preclinical/clinical training vs. conventional training (Q8 to Q10) and of (4) onsite preclinical training (Q17 to Q20), (5) participation in online theoretical courses (Q11 to Q16), (6) online degree of interactivity (Q21 to Q26), and (7) overall satisfaction (Q27 to Q32). Except for demographic data and technical availability/support, all other parts comprised multiple choice questions using a five-point Likert appreciation scale for each question (e.g., “1 = Strongly disagree”, “2 = Disagree”, “3 = Neutral/irresolute”, “4 = Agree” and “5 = Strongly agree”). A final open-ended part asking for suggestions related to the teaching approaches was also included.

### 2.3. Learning Efficiency

The protocol of a periodontal procedure—full-mouth periodontal examination—was previously taught to resident doctors during online preclinical training and written information on the subject was provided. The examination protocol evaluates six sites per tooth for probing depth, gingival recession, and clinical attachment loss according to standard clinical definitions [18] using standard equipment (dental mirror, 1 mm marking periodontal probe—UNC-15 periodontal probe, Hu-Friedy, Chicago, IL, USA). To assess the effectiveness of online teaching, resident doctors were scheduled to perform the manoeuvre on preclinical models repeatedly, three times, at three-day intervals. The working time was registered by teachers (T0, T1, T2), and the results were assessed comparatively for the two groups.

### 2.4. Statistical Analysis

Results were stored in a Microsoft Excel database. Members of the present research group reviewed the data for accuracy. Descriptive statistics were performed using Data Analysis in the Microsoft Excel spreadsheet software (Microsoft 365, Microsoft Corporation, Redmont, Washington, DC, USA) and the mean values and standard deviations (SD) for age and working times (minutes) on preclinical models (T0–T2) and the frequency of other data (e.g., gender, year of study, and the multiple-choice questions) were provided. The mean and SD were calculated for each question. Statistical analysis was performed using one-way ANOVA for repeated measures and Student’s *t*-tests for independent measures (https://www.socscistatistics.com, accessed on 3 September 2021). A *p*-value < 0.05 was considered statistically significant. Statistical analysis was conducted by one of the team members.

The comments from the third part of the questionnaire were evaluated qualitatively; more than two similar statements were grouped into categories exploring the same topics, to better understand the benefits of hybrid teaching as well as emerging needs and further steps to improve e-learning quality.

The required sample size was estimated with G-Power 3.1.9.4. The parameters used for the calculation were: type I error (0.05), type II error or power (β = 0.2, 1 − β = 0.8), type of test (two-tailed *t*-test), allocation ratio (N_control_/N_intervention_ = 1), and the predicted treatment effect (effect size, Cohen’s d = 0.575). Based on these, the computed total sample size was N = 98.

## 3. Results

### 3.1. Challenges in Periodontology Postgraduate Education Process

The COVID-19 pandemic resulted in an immediate response critical to overcome the reduced clinical training time and disrupted onsite learning in periodontology residency education. E-learning covered the theoretical curriculum and, partially, the preclinical activity by providing written instructions, visual content and video materials on specific periodontal protocols. The other part of preclinical activity was provided onsite under strict social-distancing conditions.

#### 3.1.1. E-Learning Interface

As an emergency measure, online teaching was firstly implemented in late February using the learning management System Schoology (©Schoology, New York, NY, USA) in association with Zoom (Zoom Video Communications, Inc., San Jose, CA, USA). After one month, online activity was systematically provided via the Microsoft Teams platform (Microsoft Corporation, Redmont, Washington, DC, USA) for the Faculty of Dental Medicine in Cluj-Napoca, and via Cisco Webex (Cisco Systems Inc., San Jose, CA, USA) for the Faculty of Dental Medicine in Craiova. In the case of the Faculty of Dental Medicine in Timișoara, the online activity continued through Zoom.

These platforms generously provided virtual rooms and supported screen sharing features allowing interactions of meeting participants. Special repositories were also made available.

#### 3.1.2. Challenges in Teaching and Learning

Curricular changes and teaching methods were perfected by the residency coordinators through regular meetings on the Zoom platform in order to ensure learning continuity for the residents using multiple means: courses, seminars, virtual preclinical training, and workshops through theoretical preparation; discussions; and videos on specific protocols, case presentations, conferences, webinars and repositories. Course subjects were adapted to the new pandemic context and seminars and group projects on the implications of COVID-19 on clinical activity and preventive measures were included in teaching content.

During lockdown, the teaching activity was almost exclusively provided online, except for workshops on donning and doffing personal protective equipment, which were conducted onsite under strict social-distancing conditions. Preclinical activity consisted of basic training and workshops.

#### 3.1.3. Challenges in Medical Activity

During the state of emergency declared in Romania, emergency dental settings employing university staff were organized to treat non-COVID-19 patients. Periodontology residents took part in the emergency medical activity under the strict supervision of the seniors. The dental setting functioned for six weeks (from 3 April to 15 May) based on strict preventive protocols and circuits for patients and personnel as reported previously by our team [12]. The protocols were elaborated following the reported recommendations in the literature [11,19,20] and the relevant guidelines [21,22,23]. These protocols have further been used in the current medical activity.

After lockdown, a “new” medical activity of the residents was designed, based on the same rigorous preventive protocols established during lockdown and less frequent medical interventions. In the meantime, more efficient and dense clinical time-use was implemented by training residents in preclinical settings.

#### 3.1.4. Assessment

**Figure 1:**
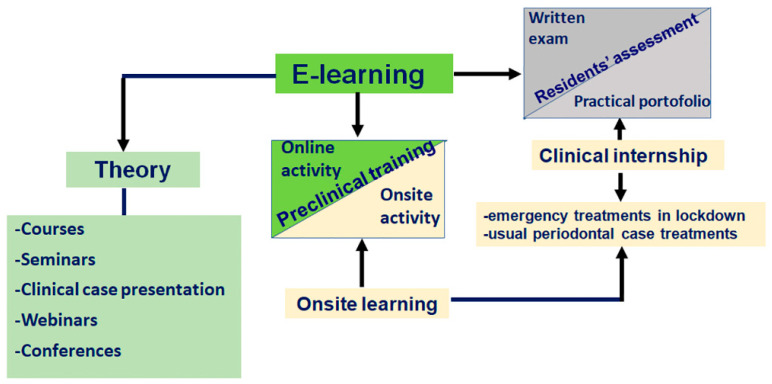
Teaching pillars in the periodontology residency program: adaptation during the COVID-19 crisis.

Formative assessment of the residents during seminars included a rigorous list of topics, running in parallel with group projects, case presentations and discussions, and reports using the medical database. A written exam at the end of the periodontology modules and the evaluation of the clinical portfolio were previewed. The clinical portfolio that validates each yearly module was revised. A summary of the activities carried out during the COVID-19 crisis is suggested in Figure 1.

### 3.2. Resident-Centred Outcomes of Teaching Activity during the COVID-19 Crisis

From 105 resident doctors registered in the three universities, only 97 matched the inclusion criteria. Out of the 97 resident doctors who completed the survey, 38 junior residents, involved in a more intensive preclinical program, were included in the PARO 1 group and 59 senior residents, more active in clinical settings, were included in the PARO 2 group.

In both groups the majority were female resident doctors, with 55.26% females enrolled in the PARO 1 group and 64.41% in the PARO 2 group. The recorded data on age indicate that most of the residents were 27 years old (26.32% in PARO 1 and 28.81% in PARO 2). The overall distribution of residents according to age is provided in Table S1.

The most common devices used for online classes (Q1, Q3) by the PARO 1 and PARO 2 groups were laptops (N = 18/47.37% and N = 38/64.41%, respectively), followed by smartphones (N = 7/18.42% and N = 12/20.34%, respectively) (Table S2).

For both the PARO 1 and the PARO 2 group, the technical issue most frequently encountered (Q2) was a poor internet connection (N = 8/21.05% and N = 25/43.1%, respectively). A significant number of residents from the PARO 1 group (39%) and from the PARO 2 group (36%) reported no technical problems associated with online learning (Table S3).

Results from the survey show that traffic restrictions during lockdown did not influence the residents’ concentration in online activities (Q4) (*p* = 0.243) or course participation (Q5) (*p* = 0.126) differently. Comparison of the results for items Q4–Q32 between the two groups are synthesized in Table S4.

Self-isolation appears to have increased the efficiency of the overall learning process (Q6) of resident doctors from the PARO 1 group in comparison with the PARO 2 group (*p* = 0.0186). Differences in the perceived influence of self-isolation on anxiety and school focus were identified between the two groups, as resident doctors from the PARO 2 group reported no perceived influence (Q7) compared with the PARO 1 group (*p* = 0.0136).

Online preclinical training of different periodontal clinical protocols vs. conventional training (Q8) was more useful and more helpful for resident doctors from the PARO 1 compared to those from the PARO 2 group, but no statistically significant differences were registered between the two groups (*p* = 0.0666).

Resident doctors from the PARO 1 group valued the efficiency of online classes more (Q9) (*p* = 0.0013) than the PARO 2 group, while resident doctors from the PARO 2 group were more critical than the PARO 1 group, reporting that the online version of clinical internships (Q10) had less than 50% the efficiency of classical onsite internships (*p* = 0.0257).

Statistically, attendance of online courses (Q11) was significantly higher for the PARO 1 group than for the PARO 2 group (*p* = 0.0178). The collected data also reveal that the PARO 1 group expressed more interest than their colleagues in continuing online learning even after resuming the normal onsite teaching program (Q12) (*p* = 0.0109) and considers online lectures a good alternative to the traditional approach (Q13) (*p* = 0.0035).

The PARO 1 group were able to focus more during online courses (Q14) (*p* = 0.002) than the PARO 2 group, while the latter got more easily distracted by other activities (housework, watching TV) during online classes (Q15), although no statistically significant differences were found between the two groups (*p* = 0.4765). In comparison with resident doctors from the PARO 2 group, the PARO 1 group declared that they had easier access to information during online lectures (Q16) (*p* = 0.0036).

Residents from the PARO 1 group considered that prior teaching and discussions on the manoeuvres during the online sessions helped them practice on study models (Q17) (*p* = 0.0089). Repeating several therapeutic manoeuvres several times on study models helped all resident doctors perform them efficiently (Q18) and improved procedure time (Q19) (*p* = 0.062 and 0.152, respectively). All residents agreed that practising the manoeuvres on study models increased their confidence in the ability to perform the manoeuvres proficiently in clinical settings (Q20) (*p* = 0.357).

Of the two groups, resident doctors from the PARO 1 group described online teaching as a more comfortable environment for asking questions during lectures (Q21) (*p* = 0.0086) and as providing them with more freedom to interact with colleagues (Q22) (*p* = 0.0349). Compared to the PARO 2 group, the opinions expressed by the PARO 1 group were better understood by their peers (Q23) (*p* = 0.0111), making them feel more comfortable during online classes (Q24) (*p* = 0.0304).

The lack of live social interaction impacted both groups (Q25), but no statistically significant difference was detected (*p* = 0.323). We also determined that audio interaction during online classes (Q26) was preferred by both PARO groups (*p* = 1).

Survey responses indicate that resident doctors were not satisfied with the clinical practice during the year of the COVID-19 pandemic (Q27), with no statistically significant differences between groups (*p* = 0.053). All participants positively valued the development of online theoretical courses (Q28) (*p* = 0.347). Both groups were found to be equally satisfied with the development of online clinical and preclinical internships (Q29) (*p* = 0.144).

Overall, the PARO 1 group seemed more open to online education (Q30) in comparison to the PARO 2 group (*p* = 0.0004). Resident doctors from both groups considered that teachers adapted promptly and demonstrated good teaching performance in online conditions (Q31) (*p* = 0.84) and were satisfied with the way the onsite preclinical internships were carried out (Q32) (*p* = 0.89).

### 3.3. Efficiency of Preclinical Learning

The full-mouth periodontal examination was demonstrated during an online teaching session and subsequently performed by resident doctors on preclinical models three times (T0, T1, T2) on different days. The attendance rate for all three training sessions was 100% for the PARO 1 group and 89.83% for the PARO 2 group.

For the PARO 1 group, the recorded mean values were T0 = 34.04 ± 10.72, T1 = 25.54 ± 8.94 and T2 = 20.54 ± 6.89. Comparison of the examination time for T0-T1, T0-T2 and T1-T2 showed statistically significant decrease regarding manoeuvre execution [(*t* = 3.745, *p* = 0.000176), (*t* = 6.526, *p* < 0.0000), and (*t* = 2.7354, *p* = 0.003897), respectively].

For the PARO 2 group, the recorded mean values were T0 = 28.72 ± 7.53, T1 = 22.52 ± 6.07, and T2 = 19.28 ± 5.37. There was a statistically significant decrease in recorded time from T0-T1, T0-T2 and T1-T2 [(*t* = 4.67, *p* < 0.0001), (*t* = 7.4825, *p* < 0.0001), and (*t* = 3.33195, *p* = 0.001195), respectively].

Intergroup comparisons were also conducted, showing that recorded time at T0 was significantly longer for the PARO 1 group (34.04 ± 10.72) than for the PARO 2 group (28.72 ± 7.53) (*t* = 2.7793, *p* = 0.006636). Conversely, the T1 values for PARO 1 (25.54 ± 8.94) and PARO 2 (22.52 ± 6.07), as well as the T2 values for PARO 1 (20.54 ± 6.89) and PARO 2 (19.28 ± 5.37), were much closer, with no statistically significant differences (*t* = 1.92521, *p* = 0.0574, and *t* = 0.97238, *p* = 0.33349, respectively).

## 4. Discussion

During the lockdown period followed by the introduction of a set of national COVID-19 restrictions, no recommendations on specific teaching modalities for medical schools were made available. As teachers, we creatively designed and employed various resources and pedagogical methods, including e-learning approaches based on literature information [24,25,26], to aid advancement and compensate for the abrupt reduction of medical activity in periodontology postgraduate training.

Institutional support has proven essential for the success of e-learning by providing technical facilities and an integrated set of strategies for improving the implementation of key skills and methods by faculty and students. All residents included in this study were offered technical support for online activities. A significant fraction of the residents included in this study did not report any technical difficulties related to online activity, regardless of the source connection used (39% from PARO 1 and 36% from PARO 2). However, only occasional problems were mentioned by the other residents. It has been reported that the lack of infrastructure, technology, internet access, and poor quality of internet services could impact both learners and faculty members [15,27,28].

In agreement with previous data [27], our findings suggest that part-time e-learning in postgraduate periodontology education was perceived as a satisfactory alternative to traditional learning. To keep residents engaged in a long-term e-learning process, the teaching staff adopted an interactive and synchronous teaching style, as recommended by specialists [14,25]. Some medical schools reported an unsatisfactory overall early experience with e-learning for medical students [27].

The two resident groups were impacted differently by e-learning, with more benefits reported by the PARO 1 group, who participated more regularly and more intensively in the online teaching and learning program than the other group. Once e-learning became a routine, the virtual activities facilitated the learning process, creating a supportive environment for increased student focus, as reported by the PARO 1 group, both regarding the theoretical aspects and the practical preclinical protocols (Q6, Q8–10, Q14, Q16, Q17). In fact, the PARO 1 group manifested their interest in continuing to use e-learning in the form of hybrid education (Q11–13), a model that would allow them to obtain the theoretical framework for the practical manoeuvres required in the clinic. In contrast, the PARO 2 residents—more involved in the clinical area and less well versed in online activities—reported a decrease in the learning efficiency associated with e-learning (Q10). Repeating protocols in online and preclinical settings increased the learning efficiency of all residents in terms of resident-centered outcomes (Q18–20).

Low-quality internet connections, a lack of social interaction, a lack of adequate practical training and difficult interactivities were cited among the limitations of e-learning in medical education [2], but the resident doctors participating in our study, especially in the PARO 1 group, seemed to have partially overcome the above-mentioned difficulties, as revealed by their answers to Q21–24. After 15 months of “new” hybrid periodontology postgraduate teaching, the organizers of the periodontology departments involved in this study strongly advocate the strengths of part-time e-learning, as also appreciated by other studies: the feasibility of “attending” the lectures; the quality of the presentations, exposed to fewer potential problems than in university halls; better concentration and, eventually; the opportunity to record lectures and access them asynchronously [2,15]; easier access to a wider variety of information that allows for a personalized learning experience [27,29]; time-saving; and the flexibility of classes [27,30]. Moreover, e-learning increases connections between residents and teachers [31]. The creation of complex electronic repositories, including videos on specific clinical subjects for enhancing learning efficiency as recognized by the literature [24,32,33,34], represented an important step in the new e-learning process in postgraduate periodontology training. The positive impact of this approach was directly visible in the overall satisfaction with online theoretical teaching (Q28, Q29) reported by both groups included in our research. Although e-learning optimizes knowledge delivery, it could hardly be imagined that virtual approaches would replace clinical traineeships. Furthermore, direct patient care is key to all dental curricula [35]. Both the PARO 1 and the PARO 2 groups seemed relatively unsatisfied with practical training (Q27), which is in agreement with the relevant literature on this topic [2,16].

Our study shows that the two groups of resident doctors perceived the educational program developed during the COVID-19 pandemic differently, and thus, based on the present results, the null hypothesis is rejected.

The periodontology departments implemented an innovative learning approach to enhancing learning efficiency during the COVID-19 pandemic and reducing contact time between patients and residents practicing within the clinic. Repetitive preclinical practice on phantoms provided training in practical skills, as resident doctors were given the possibility to perform specific manual manoeuvres based on a theoretical framework developed by the instructor, to make mistakes and learn how to overcome them [36], allowing for a significant reduction of examination duration in the meantime. This is not surprising as spaced repetition has been reported to increase the efficiency of memorization and to improve long-term retention [37].

Observation of time evolution associated with the repetition of a preclinical manoeuvre could be seen as a strong point of our study, given that chronometry is a potentially important yet understudied part of medical education [38]. Speed of execution is deemed to add value to a battery of manoeuvres by revealing potential deficiencies in technique and also by developing automaticity [39], but in a pandemic context it could also increase trainee confidence and limit clinical contact with patients.

The practice of intensive online teaching allowed us to prepare residents for preclinical approaches and for shorter but more qualitative clinical actions. We therefore believe that given the limited likelihood of resumption of medical teaching activity in the form of traditional classroom-based instruction as prior to the outbreak, it is important to validate the beneficial aspects of a “new” teaching model encompassing multiple delivery modes for further use in postgraduate training in periodontology.

An important limitation of this study is the reduced number of participants derived from the limited number of residents allocated to the three universities. Another limitation relates to the fact that only three universities were included in the study, as only these universities were assessed regarding postgraduate activity. However, this study offers valuable information about the impact of the COVID-19 pandemic on postgraduate education. The questionnaire could be used to collect information from other university centres, giving the opportunity to compare the impact of COVID-19 on postgraduate education in universities from different countries.

The current pandemic has impacted not only medical education programs, but also the medical systems through the complex pathologies and major complications induced by SARS-CoV-2. At the level of the oral cavity, the virus may cause inflammation and destruction [40] and may aggravate already existing lesions or peri-implant bone loss [41].

## 5. Conclusions

The teaching strategies used by the three Romanian periodontology departments were generally well-appreciated and commented on favourably by the residents included in our research. Part of the residents embraced the continuation of online theoretical teaching, but a large fraction also shared concerns about their clinical education and future clinical performance.

Reorganization of and further emphasis on both preclinical activity and medical practice, allowing residents to perform more accurate and efficient procedures and equipping them with more advanced clinical skills in the future, would therefore be highly important for all participants in the educational process.

The outcomes of this study highlight the benefits and the value of systematic online e-learning approaches, recognized as a key component of the new hybrid teaching approach, a methodology that could probably be maintained over the medium-term and beyond.

## Data Availability

The datasets generated during the current study are available from the corresponding author on reasonable request.

## Supplementary Materials

The following supporting information can be downloaded at: https://www.mdpi.com/article/10.3390/ijerph19084488/s1, Table S1: The overall distribution of residents according to age; Table S2: Devices used by resident doctors during online study; Table S3: Technical problems associated with online learning; Table S4: Significance of the differences between evaluated parameters.

## Appendix A. Survey Used for the Present Study, Translated into English for the Purposes of the Study

**No.**	**Part**	**Question**
1.	Technical availability	(1) I had sufficient technical means to participate in courses (2) The most common technical problems that affected my good participation in courses were related to: internet connection, PC, laptop, tablet, smartphone, microphone, webcam, other (3) The most frequently used device to participate in courses was: PC, laptop, tablet, smartphone, other
2.	Impact of traffic restrictions and self-isolation	(4) Traffic restrictions and social distancing had an impact on my concentration in classes (5) I would have attended the courses to the same extent even if the traffic restrictions during the state of emergency had not been in force (6) Self-isolation has increased the efficiency of my overall learning process (7) Self-isolation created a state of anxiety and affected my concentration in school
3.	Online preclinical/clinical training vs. conventional training	(8) They were useful and helped me better understand the subject (9) The online version had the same learning efficiency as the conventional version (10) The efficiency of the online version of clinical internships was below 50% compared to conventional onsite internships
4.	Participation in online courses	(11) My attendance in online courses was better than usual (12) Upon return to a conventional learning format, I would like to participate in online courses (13) I consider online courses a good alternative to classic courses (14) I think I would focus better during online courses than during conventional ones (15) I was more easily distracted by other activities (housework, watching TV, etc.) during online courses than I was during conventional classes (16) I think I had better access to information during online courses (I could hear better, I could see better what was being taught, I was not distracted by the presence of colleagues in the amphitheater)
5.	Onsite preclinical training	(17) Teaching and discussing manoeuvres during online courses and internships helped me execute them on study models, during onsite preclinical internship (18) Repetitive practice of the manoeuvres on a study model during onsite preclinical training helped me to learn those manoeuvres (19) Repetitive practice of the manoeuvres on a study model during onsite preclinical training helped me increase individual manoeuvre execution speed (short working time) (20) Repetitive practice of the manoeuvres on a study model during onsite preclinical training gave me confidence that I would be able to perform them correctly and efficiently in the clinic.
6.	Online interactivity	(21) I feel more comfortable asking questions during online courses than during regular courses (22) The online environment gives me more freedom to interact with colleagues and teachers (I can share my screen, for example.) (23) The views I expressed in class were well understood and appreciated by other colleagues (24) I feel more comfortable attending online classes than regular ones (25) The fact that I don’t physically meet my colleagues bothers me (26) I feel comfortable about audio interaction with other colleagues during the online course discussions.
7.	Overall satisfaction	(27) I am satisfied with the clinical practice I carried out during the year of the pandemic (28) I am satisfied with the way online courses were conducted (29) I am satisfied with the way online clinical and preclinical internships were conducted (30) Overall, I can say that I prefer online education to traditional education (31) I consider that the teaching staff has adapted well to the online environment and taught the subject efficiently (course materials, case presentations, interactive topics) (32) I am satisfied with the way onsite preclinical internships were conducted.
