# Supplementary material for: The Impact of the COVID-19 Pandemic on Romanian Postgraduate Periodontal Residency Teaching: Past Experience, Present Imperatives and Future Considerations in a Multicentric Evaluation
Source: Int J Environ Res Public Health. 2022 Apr 8;19(8):4488. doi: 10.3390/ijerph19084488 (PMC9031164; doi:10.3390/ijerph19084488)
Supplement: Supplementary file 1 [file ijerph-19-04488-s001.zip › ijerph-1589182-supplementary.pdf]

Table S1. The overall distribution of residents according to age.

Age	PARO 1	PARO 2
26	6(15.79%)	7(11.86%)
27	10(26.32%)	17(28.81%)
28	7(18.42%)	12(20.34%)
29	6(15.79%)	6(10.17%)
30	1(2.63%)	9(15.25%)
31-40	7(18.42%)	5(8.47%)
over 40	0	3(5.08%)

Table S2. Devices used by resident doctors during online study

Used Device	PARO 1	PARO 2
Laptop	18(47.37%)	38(64.41%)
Laptop, Mobile phone	4(10.53%)	2(3.39%)
PC	2(5.26%)	3(5.08%)
PC, Laptop, Mobile phone	2(5.26%)	1(1.69%)
PC, Mobile phone	3(7.89%)	1(1.69%)
Tablet, Mobile phone	2(5.26%)	1(1.69%)
Mobile phone	7(18.42%)	12(20.34%)
Laptop, Tablet	0	1(1.69%)

Table S3. Technical problems associated with the online learning.

Technical Issues	PARO 1	PARO 2
Internet connection	7(18.42%)	25(43.1%)
Internet connection, Microphone/Webcamera	3(7.89%)	2(3.45%)
Internet connection, No problems	1(2.63%)	
Internet connection, PC/Laptop	4(10.53%)	
Internet connection, PC/Laptop, Microphone/Webcamera	1(2.63%)	
Internet connection, PC/Laptop, Microphone/Webcamera, Tablet, Mobile phone	1(2.63%)	
Microphone/Webcamera	2(5.26%)	5(8.62%)
PC/ Laptop	3(7.89%)	3(5.17%)
Mobile phone	1(2.63%)	2(3.45%)
Sound	1(2.63%)	
No problems	15(39.47%)	21(36.21%)

Table S4. Significance of the differences between evaluated parameters

Question	t- test	p	Mean value PARO 1	Mean value PARO 2
4	1.1749	0.243	2.34	2.66
5	1.5415	0.1265	4.16	3.8
6	2.3951	0.0186	3.11	2.51
7	-2.5149	0.0136	2.18	2.86
8	1.8558	0.0666	3.32	2.86
9	3.308	0.0013	2.76	2
10	-2.2667	0.0257	3.16	3.71
11	2.411	0.0178	3.29	2.64
12	2.5981	0.0109	3.5	2.83
13	2.9945	0.0035	3.39	2.59
14	3.8966	0.0002	3.03	2.12
15	0.7147	0.4765	3.18	3.39
16	2.982	0.0036	3.13	2.37
17	2.674	0.0089	3.58	2.9
18	1.8886	0.0623	3.97	3.47
19	1.4442	0.152	4.08	3.69
20	0.0241	0.3578	3.84	3.59
21	2.6817	0.0086	3.13	2.49
22	2.1407	0.0349	2.89	2.36
23	2.5895	0.0111	3.47	3
24	2.1982	0.0304	3.18	2.61
25	0.9927	0.3234	3.46	3.76
26	1	1	3	3
27	1.9553	0.0538	2.68	2.17
28	0.9449	0.3471	4.05	3.85
29	1.4725	0.1442	3.71	3.36
30	3.6581	0.0004	2.58	1.71
31	0.1938	0.8468	4.08	4.03
32	0.1383	0.8903	4	4.03
